# A novel method to discover fluoroquinolone antibiotic resistance (qnr) genes in fragmented nucleotide sequences

**DOI:** 10.1186/1471-2164-13-695

**Published:** 2012-12-11

**Authors:** Fredrik Boulund, Anna Johnning, Mariana Buongermino Pereira, DG Joakim Larsson, Erik Kristiansson

**Affiliations:** 1 Department of Mathematical Sciences, Chalmers University of Technology and University of Gothenburg, Göteborg, SE-412 96, Sweden; 2 Institute of Neuroscience and Physiology, the Sahlgrenska Academy at the University of Gothenburg, Box 434, Göteborg, SE-405 30, Sweden; 3 Department of Infectious Diseases, Institute of Biomedicine, the Sahlgrenska Academy at the University of Gothenburg, Box 434, Göteborg, SE-405 30, Sweden

**Keywords:** Metagenomics, Antibiotic resistance, Fluoroquinolones, PMQR, Qnr, Hidden markov models

## Abstract

**Background:**

Broad-spectrum fluoroquinolone antibiotics are central in modern health care and are used to treat and prevent a wide range of bacterial infections. The recently discovered *qnr* genes provide a mechanism of resistance with the potential to rapidly spread between bacteria using horizontal gene transfer. As for many antibiotic resistance genes present in pathogens today, *qnr* genes are hypothesized to originate from environmental bacteria. The vast amount of data generated by shotgun metagenomics can therefore be used to explore the diversity of *qnr* genes in more detail.

**Results:**

In this paper we describe a new method to identify *qnr* genes in nucleotide sequence data. We show, using cross-validation, that the method has a high statistical power of correctly classifying sequences from novel classes of *qnr* genes, even for fragments as short as 100 nucleotides. Based on sequences from public repositories, the method was able to identify all previously reported plasmid-mediated *qnr* genes. In addition, several fragments from novel putative *qnr* genes were identified in metagenomes. The method was also able to annotate 39 chromosomal variants of which 11 have previously not been reported in literature.

**Conclusions:**

The method described in this paper significantly improves the sensitivity and specificity of identification and annotation of *qnr* genes in nucleotide sequence data. The predicted novel putative *qnr* genes in the metagenomic data support the hypothesis of a large and uncharacterized diversity within this family of resistance genes in environmental bacterial communities. An implementation of the method is freely available at http://bioinformatics.math.chalmers.se/qnr/.

## Background

Antibiotics are one of our most powerful tools for treating and preventing bacterial infections and have since their introduction vastly improved human health and drastically reduced mortality rates. The high use of antibiotics in human and veterinary medicine has however resulted in an accelerated development of multiresistant bacteria [1,2]. Bacteria can adapt to an antibiotic selection pressure by altering their genome, either by mutations in pre-existing DNA or through the acquisition of resistance genes [3]. Since resistance genes can be horizontally transferred between bacterial cells, antibiotic resistance can rapidly spread within and between bacterial communities [4-6]. Many types of antibiotics are derived from compounds that are naturally found in the environment and bacteria have developed resistance genes as a protection mechanism. Environmental bacterial communities have therefore been hypothesized to contain a large and unexplored collection of antibiotic resistance genes [7-10]. Antibiotic resistance genes were present in environmental bacterial communities long before they emerged in human pathogens [11]. As a consequence, many of the antibiotic resistance genes found in clinical settings have been horizontally transferred from environmental bacteria [12,13].

The broad-spectrum fluoroquinolone antibiotics were introduced in the early 1960’s and are today extensively used in human and veterinary medicine. Fluoroquinolones interacts with the essential bacterial type II topoisomerases (topoisomerase IV and DNA gyrase) and thereby inhibits DNA replication. The most effective fluoroquinolone resistance mechanism is chromosomal mutations in the antibiotic target proteins which confers high levels of resistance in several bacterial species [14,15]. Recently, a family of mobile fluoroquinolone antibiotic resistance genes called *qnr* was discovered [16,17]. These mobile plasmid-mediated quinolone resistance genes (sometimes labeled PMQR) have been grouped into five recognized classes; *qnrA*, *qnrB*, *qnrC*, *qnrD*, and *qnrS* and it is currently unknown whether more classes exist. The *qnr* genes encode proteins that prevent fluoroquinolones from interacting with DNA/type-II-topoisomerase complexes formed during DNA replication, thus preventing fluoroquinolone inhibition. The levels of resistance conferred by *qnr* genes are generally lower than chromosomal mutations but can reach up to 1 mg/L (minimum inhibitory concentration) depending on the organism and specific antibiotic compound [18].

The *qnr* genes belong to the larger family of pentapeptide repeat proteins (PRP), which are ubiquitously present with more than 500 variants described in all forms of life [19]. All PRPs are characterized by a sequence feature consisting of repeating subunits of five amino acid residues following the form A(D/N)LXX. This repetitive pattern makes PRPs fold into a β-helix that performs a wide range of cellular functions and they are found both membrane bound and in the cytoplasm [20]. For *qnr* genes the β-helix resembles the structure of the DNA spiral and interacts with type II topoisomerases and thereby prevent fluoroquinolone antibiotics to inhibit the function of the complex [21,22]. Despite the strong similarity in the repeating amino acid pattern between *qnr* sequences and other PRPs it is unclear exactly why *qnr* genes provide resistance to fluoroquinolones.

Further characterization of *qnr* genes is necessary to fully understand their function and estimate their diversity. Assuming the presence of antibiotic resistance genes in clinical settings is the result of transfer of mobile genetic elements from the environment, it is natural to search environmental microbial communities to find previously unidentified *qnr* genes. Recent culture-independent methods such as metagenomics enables unprecedented exploratory analysis of the genetic basis in microbial communities [23,24]. This is especially true considering that more than 99% of environmental bacterial communities do not submit easily to cultivation and would consequently be missed with sampling and analysis of individual strains [25,26]. In combination with next-generation DNA sequencing technologies metagenomics provide means for culture-independent studies of bacterial communities at a very high resolution. However, high-throughput sequencing equipment can currently only produce short DNA fragments (typically 75-400 nucleotides long) which substantially limits the sensitivity and specificity of identifying genes such as *qnr*[27].

*In-silico* approaches have previously been used to identify novel variants of *qnr* genes. For example, Fonseca *et al.* identified *qnr*VC1 and *qnr*VC2 in *Vibrio cholerae* using sequence comparison to existing plasmid-mediated *qnr* genes [28]. A similar approach was used by Sanches *et al.* to identify several chromosomal *qnr* variants, including multiple members of the class *Smqnr* from *Stenotrophomonas maltophilia*[29]*,* and by Velasco *et al.* to discover *Smaqnr* in *Serratia marcescens*[30]. However, all of these studies used sequence alignment tools such as BLAST which do not explicitly make use of the repetitive structure of the *qnr* genes. Furthermore, none of the previous suggested methods were adapted to short sequence lengths and high volumes of data which makes them inapplicable to sequences from shotgun metagenomics.

In this paper, we describe a novel method to identify fluoroquinolone antibiotic resistance genes in DNA sequence data. By using hidden Markov models combined with a length-dependent classification rule, the method is able to discriminate between *qnr* and other pentapeptide repeat proteins not associated with a resistance phenotype. Cross-validation estimated that the method had a high statistical power of detecting fragments of *qnr* genes in metagenomic data, even at fragment lengths as short as 100 nucleotides. The method was applied to sequence data from various databases and both known and novel putative *qnr* genes were identified. An implementation of the method is freely available at http://bioinformatics.math.chalmers.se/qnr/.

## Results

**Figure 1:**
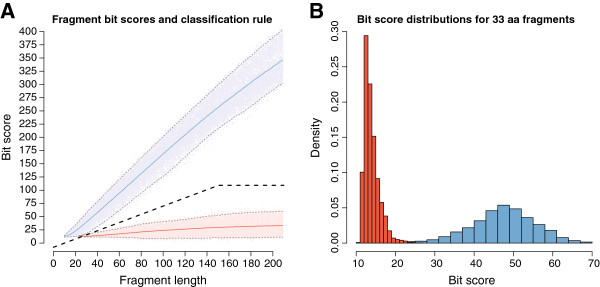
**Fragment bit scores and classification rule. A**) The figure shows the distribution of the fragment bit scores at different fragment lengths. The separation between the *qnr* fragments (light blue) and non-*qnr* fragments (light red) increase for longer fragment lengths. The solid blue and red lines show the average bit scores for *qnr* and non-*qnr* fragments, with their 99th and 1st percentiles in grey dashed lines above and below, respectively. The thick dashed line in black shows the classification function with the optimized parameters K=0.778, M=-7.89, D=150.64 [see Additional file 1: Figure S1, Additional file 2: Figure S2, Additional file 3: Figure S3, Additional file 4: Figure S4, Additional file 5: Figure S5 for plots corresponding to each separate class of *qnr*]. **B**) The bit scores when compared to the hidden Markov model for 33 amino acid long fragments, corresponding to the approximately 100 nucleotides long sequence reads common in next-generation sequencing technologies. At this fragment length, the *qnr* fragments (blue) are clearly separated from the non-*qnr* (red) with only a small overlap.

A hidden Markov model (HMM) was constructed from a multiple sequence alignment of all currently known and experimentally verified plasmid-mediated *qnr* resistance gene amino acid sequences [31]. Using the database search software HMMER3, we analyzed the empirical bit score distributions produced by applying the HMM to two sources of protein sequence data; a*)* true *qnr* fragments, created from randomly fragmented *qnr* sequences and *b)* non-*qnr* fragments, created from pentapeptide repeat protein (PRP) sequences not associated with a fluoroquinolone resistance phenotype (see Methods). To visualize the bit score distributions of fragmented sequences, random fragments of *qnr* and non-*qnr* sequences were created for each fragment length between 10 and 210 amino acid residues (i.e. full length *qnr* sequences) and their scores against the HMM were plotted as a function of fragment length. As indicated by Figure 1 true *qnr* fragments had bit scores that were approximately linear in relation to their fragment length while the bit score distribution of the non-*qnr* fragments was centered around 33. A two-part linear classification function was therefore introduced to discriminate between true *qnr* and non-*qnr* fragments. For fragments up to a length threshold (D), the classification function was linear with an intercept M and slope K. For fragments longer than D, the function used a fixed cutoff *C* = *K* × *D* + *M* (Figure 1A, Additional files 1, 2, 3, 4, 5).

Cross-validation was used to optimize the parameters M, K and D of the classification function for identification of novel classes of *qnr* genes. The optimization was performed for five different models where each model was created by excluding one class of plasmid-mediated *qnr* proteins (i.e. QnrA, QnrB, QnrC, QnrD and QnrS). The cross-validation was then performed using disjoint set of fragments, one for parameter estimation (training) and one for evaluation of the corresponding performance (validation). The training and validation data sets were created from fragments of both *qnr* genes and non-*qnr* PRP genes without any associated resistance phenotype. For each of the five models, the excluded *qnr* class was also removed from the training dataset. The corresponding performance was, on the other hand, evaluated only using the excluded *qnr* class and a set of non-*qnr* genes. Thus, the ability to classify novel *qnr* genes was evaluated on fragments of gene classes not included in the model. The cross-validation was performed with random fragments ranging from 10 to 209 amino acid residues, each length repeated 2500 times. The parameters of the classification function were estimated to M = -7.89 (1.37), K = 0.778 (0.084), D = 150.64 (27.05) (average over all five models, standard deviation in brackets). The corresponding fixed cutoff C was calculated to C = 109.64 (16.40).

**Figure 2:**
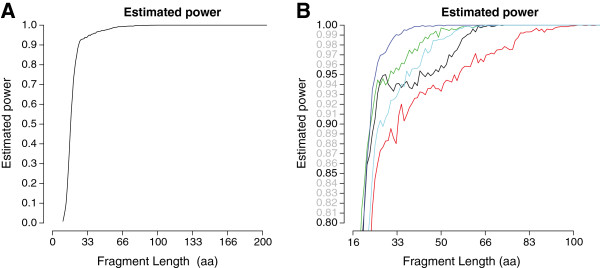
**Estimated power. A**) The figure shows the estimated power of detecting fragments from novel classes of *qnr* as a function of fragment length in nucleotides (averaged over the five different models used in the cross-validation). At a fragment length of 33 amino acids (approximately 100 nucleotides), the power to detect fragments from novel classes of *qnr* genes was estimated to 94% which increased to 100% for 100 amino acid long fragments. **B**) A magnification of the upper left region showing the power of detecting each class of *qnr* genes: QnrA (black), QnrB (red), QnrC (green), QnrD (dark blue) and QnrS (cyan). Corresponding plots for the specificity are available as [Additional file 6: Figure S6].

The optimized classification function parameters were then used to validate the statistical power to detect novel putative fragments. At a fragment length as short as 33 amino acids the average power for correctly classifying fragments from novel putative *qnr* gene classes was 94% (Figure 2A). The results differed between the five models (Figure 2B): for a 33 amino acid long sequence, the power to identify a QnrD fragment (given a model built from QnrA, B, C and S) was highest (99.04%) while the power of identifying a QnrB fragment (given QnrA, C, D and S) was the lowest (88.02%). The specificity was estimated to be above 99.27% for all models and all fragment lengths [Additional file 6: Figure S6]. See Methods for full details.

To search for novel putative *qnr* gene variants a model based on all five classes of plasmid-mediated *qnr* genes together with the classifier with the optimized parameter values was applied to protein sequences from various databases and metagenomic sequencing projects; GenBank [32], CAMERA [33], MG-RAST [34], contigs from Meta-HIT [35], and several data sets from SRA [36] (see Table 1 and Methods). A smaller metagenomic dataset from a recent study where a high abundance of *qnr* genes was detected was also included [37]. The total number of fragments available in all datasets was 478,025,600 comprising 214,168,682,742 nucleotides. In total, 1733 (3.6 × 10^-4^%) sequence fragments classified as *qnr* by the method. For the metagenomes the proportion of *qnr* fragments was estimated to 2.8 × 10^-4^% (1275 out of 463,364,852 metagenomic fragments), reflecting the low abundance of *qnr* genes in the environment. All fragments that classified as *qnr* were stringently clustered into 475 groups (where 165 contained more than one fragments) and annotated against GenBank and the list of known *qnr* genes [31] [Additional file 7: Table S1]. Among these clusters, all five classes of plasmid-mediated *qnr* were represented as well as 28 previously described chromosomally located variants [28,29,38-41]. In addition, one contig in group #1, which consisted entirely of metagenomic fragments, represented a full length sequence of a novel putative *qnr* gene with 93% identity (97% similarity) to QnrB1. During the course of this project this sequence was accepted as a novel QnrB variant, QnrB35, and submitted to GenBank [GenBank:AEL00456] [39]. The method was hence capable of reconstructing complete *qnr* sequences directly from fragmented metagenomic data. This was particularly evident since the complete sequence of QnrB35 was not available in any of the datasets at the time of their retrieval in this project.

**Table 1:** **Data sources searched for *qnr *gene fragments**

**Data source**	**Number of sequences**	**Number of nucleotides *(approximate)***	**Identified putative *qnr *fragments**
CAMERA [33]	161,016,287	57,118,358,119	217
GenBank (nt) [32]	14,627,404	35,003,500,149	392
GenBank (env_nt) [32]	18,438,927	7,602,413,875	54
GenBank (refseq) [32]	33,074	7,192,954,783	66
Meta-HIT [35]	6,589,348	10,322,657,198	2
MG-RAST [42]	74,767,763	29,132,992,517	226
SRA [36]	202,090,286	67,627,717,961	516
India Patancheru [37]	462,241	168,088,140	260
*Total:*	*478,025,600*	*214,168,682,742*	*1733*

The method discovered 732 fragments of metagenomic origin that clustered in 440 groups which did not contain any of the previously described plasmid-mediated or chromosomal *qnr* genes. An additional 11 sequences of novel putative *qnr* genes in the genomes of 9 sequenced bacteria were also discovered [Additional file 7: Table S1]. Table 2 shows five examples of groups containing sequences classified as novel putative *qnr* genes by the method. Sequence #1 was constructed from fragments originating from baby stool metagenomes [43] [SRA accession SRX032366] and shared 79% sequence identity with QnrB37. Sequence #2 was discovered in an environmental samples from coastal sea water outside the North American coast [MG-RAST accession 4441580] as a part of the Gene Ocean Sampling Expedition [44]. This sequence is a 218 amino acid long fragment that shares 33% sequence identity with QnrC. The next three sequences were discovered in bacterial genomes in GenBank. Sequence #3 was discovered in the chromosome of *Dickey dadantii* 3937 [GenBank:NC_014500.1] and was a 213 amino acid long sequence with 68% identity to QnrB28. Sequence #4 was found in the chromosome of *Xenorhabdus bovienii* [GenBank:NC_013892.1] and was a 211 amino acid long sequence with 66% identity to QnrB19. Sequence #5 came from the chromosome of *Vibrio furnissii* [GenBank:CP002378.1] and was a 218 amino acid long sequence sharing 72% identity with QnrC. Full results, including all 475 groups and their annotation, are available in [Additional file 7: Table S1].

**Table 2:** **Examples of identified novel putative *qnr *sequences**

**Example #**	**Group**	**Source(s)**	**Contig length (aa)**	**Model bit score**	**Most similar plasmid-mediated*qnr***
1	1	Metagenome:	214	356.9	QnrB37 (79% identity)
		SRA: SRX032366			
2	12	Metagenome:	218	131.2	QnrC (33% identity)
		MG-RAST: 4441580			
3	78	Chromosome:	213	326.4	QnrB28 (68% identity)
		Dickeya dadantii 3937, NC_014500.1			
4	81	Chromosome:	211	294.6	Qnr19 (66% identity)
		Xenorhabdus bovienii SS-2004, NC_013892.1			
5	199	Chromosome:	218	350.0	QnrC (72% identity)
		Vibrio furnissii, CP002378.1			

## Discussion

Qnr genes provide resistance to broad-spectrum fluoroquinolone antibiotics and can move between bacteria using horizontal gene transfer. However, the total number of *qnr* classes and their diversity in environmental bacterial communities is not clear. We therefore developed a novel method to identify new classes of *qnr* genes in fragmented metagenomic data. The method uses a hidden Markov model (HMM) to identify candidate *qnr* fragments which are then further classified based on their model score and sequence length. Cross-validation confirmed that the method had a high sensitivity and specificity to detect fragments from novel classes of known *qnr* genes, even at fragments as short as 33 amino acid residues. This makes the method applicable to many forms of nucleotide data, including sequences generated by next-generation DNA sequencers. From public sequence repositories the method classified 1733 sequence fragments (3.6 × 10^-4^%) as *qnr*, which were further clustered into 475 groups. The method also identified 39 chromosomal *qnr* variants in 33 bacterial species.

Several of the novel putative *qnr* genes identified in this study have to the authors’ best knowledge previously not been described in literature. Experimental verification, including phenotypic profiling in multiple bacterial hosts, is therefore necessary to fully evaluate the resistance potential of our predictions. However, the cross-validation demonstrated that the proposed method had a high sensitivity and could discriminate between fragments from classes of known *qnr* and pentapeptide repeat proteins without a resistance phenotype (Figure 2A). The method was also able to identify all previously reported classes of *qnr* genes, including the variant *qnr*B35 which was at the time for this analysis not submitted to the database and thus not included in the hidden Markov model. This shows that the method has a high predictive power and it is therefore possible that several of the predictions indeed represent previously unidentified novel classes or variants of *qnr* genes.

Many of the identified putative *qnr* gene fragments were discovered in metagenomes sampled from different types of environments, e.g. human gut [43], seawater [44] and river sediment [37] [see Additional file 7: Table S1]. This indicates that there is an unexplored diversity of *qnr* genes within environmental bacterial communities and that these can be identified by metagenomic sequencing. However, the amount of nucleotide data currently represented in the sequence repositories merely reflects a tiny fraction of the total microbial diversity on earth [45-47]. In addition, the estimated relative abundance of unknown fragments from putative *qnr* genes was 2.8 × 10-4% (1275 out of 463,364,852 metagenomic fragments) underlining the vast amounts of sequence data needed to identify and assemble *qnr* genes from environmental data. It is therefore possible, and even likely, that there are additional variants of *qnr* genes present in the environmental bacterial communities currently not represented in the sequence repositories due to the heavy undersampling. The data that is currently being generated by large-scale reference metagenome projects, such as the Earth Microbiome Project [48] and the Gene Ocean Sampling [44], will offer a substantially higher sequencing depth and may therefore reveal additional classes and variants of *qnr* genes.

Our results show that hidden Markov models are highly suitable for identifying sequence fragments from *qnr* genes. The model used in this study was derived from a multiple alignment of *qnr* genes and can thereby infer information on the degree of variability at different amino acid positions in the sequence [49]. This is especially useful for pentapeptide repeat proteins which generally have a low sequence similarity except in the conserved residues of the distinctive repetitive A(D/N)LXX motif. In contrast, traditional sequence alignment tools such as BLAST cannot distinguish between important variation in the repeating pattern and variation in the intermediate regions. Previous methods to identify novel *qnr* genes from DNA sequence data have used BLAST and may therefore have limited sensitivity and specificity [49]. The proposed method has, on the other hand, demonstrated a high power of detecting new classes of *qnr* genes (Figure 2) and is hence a more suitable approach for identification and annotation of *qnr* genes.

Controlling the number of false predictions is vital for large-scale data analysis. A low specificity can generate a massive amount of false positives and thereby decrease the quality of analysis and the biological interpretation of the downstream results (in this case the sequence groups). Based on the distribution of bit scores for the sequence fragments (Figure 1A) it is clear that a traditional cut-off would not be sufficient to discriminate between *qnr* and non-*qnr* PRPs with both a high sensitivity and specificity. Indeed, a single bit score cut-off would have to be set to 75 to minimize false positives across all fragment lengths, effectively removing the ability to classify fragments shorter than 100 amino acid residues (300 nucleotides). Instead, a linear classification function dependent on fragment length for short fragments enabled correct identification while maintaining a high specificity [Additional file 6: Figure S6]. This makes the method suitable for analysis of large datasets consisting of short sequence fragments and the method is therefore directly applicable to data from next-generation sequencing technologies such as Illumina’s sequencing by synthesis, Life Technologies’ sequencing by ligation (SOLiD) or Roche’s 454 pyrosequencing [50].

The hidden Markov model used by the method was created from all known plasmid-mediated *qnr* genes with experimentally validated resistance phenotype. However, several recent studies have described chromosomally located *qnr* genes in wide range of species (e.g. *Vibrio spp. alginolyticus*, *Vibrio harveyi* and *Aeromonas hydrophilia*) [28,29,38,40,41]. These chromosomal *qnr* genes show a relatively high sequence similarity to their plasmid-mediated relatives and some have been shown to confer resistance towards fluoroquinolones when expressed in *E. coli* (e.g. SmaQnr and SmQnr) [29,41]. Their potential to transfer horizontally between bacteria is however not clear. Even though the hidden Markov model was based on plasmid-mediated gene variants, the method demonstrated a high sensitivity to detect *qnr* genes in bacterial chromosomes. In fact, the method identified 28 previously reported chromosomally located *qnr* genes in 24 species. In addition, 11 potentially novel chromosomal *qnr* genes in 9 different species were also identified [Additional file 7: Table S1]. Interestingly, four previously suggested chromosomal *qnr* genes were not classified as such by the method. These genes, which are located in *Alkaliphilus metalliredigens, Bacteroides thetaiotaomicron, Bacillus weihenstephanensis* and *Anabaena variabilis* have previously been identified as putative *qnr* genes using BLAST [38]. However, all these genes share low sequence similarity to other *qnr* genes and their resistance phenotype has so far not been validated. The four genes received very low scores by our model, which may indicate that these are false predictions and hence not *qnr* genes. All other previously described *qnr* genes received high scores by the model and were thus classified as *qnr*.

The method described in this study has been implemented as a freely available application in Python. The application searches any specified sequence dataset, classifies the matching sequences as *qnr* or non-*qnr* and clusters the results into groups of putative *qnr* genes [see Additional file 8: Figure S7 for an overview]. The implementation is straightforward to use, has been optimized to handle data sizes of the order of terabytes, and is suitable for use on standard desktop computers. The package is documented with internal functions thoroughly commented in the distributed source code, making it possible to interface them directly from related applications. The application can be installed and run on any modern GNU/Linux system and it is available from http://bioinformatics.math.chalmers.se/qnr/.

## Conclusions

In this study we proposed a new method to detect and annotate novel classes of *qnr* antibiotic resistance genes in nucleotide sequence data. The method uses a hidden Markov model with a fragment length-dependent classification rule and has a high sensitivity and specificity, even for sequences as short at 100 nucleotides. This makes the method directly applicable to the immense amount of data generated by the next-generation DNA sequencing techniques. Based on sequence data currently available in the repositories, the method was able to identify all previously reported plasmid-mediated *qnr* genes as well as the vast majority of the previously reported chromosomal variants. In addition, the method predicted several novel putative *qnr* genes and some of these were discovered in shotgun metagenomes, which may indicate a large and unknown diversity of *qnr* genes in uncultured environmental bacteria.

## Methods

A hidden Markov model was based on a multiple sequence alignment of sequences from the reference list of acknowledged and experimentally verified plasmid-mediated *qnr* genes [31]. Peptide *qnr* sequences were aligned using MAFFT [51] with default settings. The alignment quality was manually assessed and then used as input for the construction of the hidden Markov model using HMMER3 [49] with default settings.

Investigation of the empirical bit score distribution of the HMM was performed by drawing random fragments of both *qnr* and non-*qnr* genes (Figure 1). This led to the creation of a classifier consisting of a two-part linear discrimination function using information of fragment length (L_f_) and fragment bit score (S_f_) from the hidden Markov model from HMMER. The classifier was defined by three parameters; linear intercept (M), linear slope (K), and long fragment definition (D). A fragment with length, L_f_, and domain bit score, S_f_, was classified as *qnr* if L_f_ < D and S_f_ ≥ K  ×  L_f_ + M, or if L_f_ ≥ D and S_f_ ≥ K  ×  D + M.

Cross-validation was used to estimate the parameters and to evaluate the performance of the model. Five different models were created and for each model one class of plasmid-mediated *qnr* genes was excluded. Two different kinds of sequences were used in the cross-validation: true *qnr* genes and non-*qnr* pentapeptide repeat protein sequences. The source of true *qnr* sequences was the reference list of *qnr* sequences [31] and the source of non-*qnr* sequences was sequences from GenBank annotated as pentapeptide repeat proteins (PRP) with the COG1357 annotation, but without a known resistance phenotype.

Two sets of data were created for each model in the cross-validation; a training and a validation set. The training sets consisted of a combination of true *qnr* sequences excluding the class which was left out from the model in question and a set of 90 random non-*qnr* genes. The validation sets contained all known variants of the previously excluded *qnr* class plus a different set of 421 non-*qnr* genes. For example, the first model was based on all known plasmid-mediated *qnr* sequences excluding the sequences from the class *qnrA*. This model was then applied to training data consisting of true *qnr* sequences excluding *qnrA* and a set of non-*qnr* genes. The classification function was then applied to validation data consisting exclusively of *qnrA* and a different set of non-*qnr* sequences where the performance of the model to identify unknown (i.e. novel) classes of plasmid-mediated *qnr* was estimated. The fragments used in the cross-validation were randomly generated from the training and validation data sets for each model by randomly drawing a *qnr*/non-*qnr* fragments with equal probability. For each dataset, 2500 random fragments were created for each fragment length between 10-210 amino acids. A relatively high mutation rate on amino acid sequence was added by randomly substituting each residue for another with the probability of 5% to introduce a substantial amount of noise.

Parameter values for the classification function were optimized using particle swarm optimization where the parameter spaces for the three parameters were explored (ranges in brackets): M [-20, 30], K [0, 2], and D [30, 210]. Optimization was performed six times using a swarm size of 30 particles with 50 iterations in each run with randomized starting points in parameter space. The objective was to achieve a high true positive rate (TPR) without letting the false positive rate (FPR) becoming too high. The objective function was therefore set to TPR-FPR. The statistical power of the model for identifying novel plasmid-mediated *qnr* gene variants was computed by using the average parameter values from the six optimization runs when applying the model to the validation data sets (Figure 2).

The nucleotide datasets used in this project (Table 1) were public sequence data sets downloaded in April 2011 (GenBank version 183). Data from the NCBI Sequence Read Archive (SRA) was selected using the search string “metagenom* AND (454 AND (flx OR titanium)) NOT 16S NOT V6 NOT V9” which generated 1756 hits at the time data was sourced for this project. The sequence data was first translated into all six reading frames using bacterial translation table 11 in EMBOSS *transeq*[52]. The translated sequences were fed into HMMER3 program *hmmsearch* to find hits against the model. The only non-default settings used were --notextw and --cpu 8, with no change from default settings for inclusion or reporting thresholds. All hits discovered by HMMER3 were instead subjected to the classification function and hits that classified as *qnr* were clustered using Blastclust [53]. Clustering parameters used were fragment similarity threshold 90% and minimum length coverage 25%. Cluster groups (containing hits/sequence fragments) were aligned using MAFFT to produce overlapping multiple alignments. The aligned groups were then manually adjusted to identify overlapping fragments that formed longer contigs and complete *qnr* gene contigs. Finally, such contig sequences were annotated using a combination of the reference *qnr* compilation [31] and the GenBank data displayed in Table 1.

## Competing interests

The authors declare no competing interests.

## Authors’ contributions

FB, EK and AJ planned the project. FB developed and implemented the method, performed the cross-validation, interpreted the clustering results and annotated the hits. MBP assisted with the implementation of the method. FB and EK drafted the manuscript. The work was supervised by EK, AJ and DGJL. All authors read and approved the final manuscript.

## Supplementary Material

Additional file 1 **Figure S1.** Fragment bit scores with HMM constructed without QnrA. Bit scores of fragments against the hidden Markov model where all sequences from QnrA were excluded. Click here for file

Additional file 2 **Figure S2.** Fragment bit scores with HMM constructed without QnrB. Bit scores of fragments against the hidden Markov model where all sequences from QnrB were excluded. Click here for file

Additional file 3 **Figure S3.** Fragment bit scores with HMM constructed without QnrC. Bit scores of fragments against the hidden Markov model where all sequences from QnrC were excluded. Click here for file

Additional file 4 **Figure S4.** Fragment bit scores with HMM constructed without QnrD. Bit scores of fragments against the hidden Markov model where all sequences from QnrD were excluded. Click here for file

Additional file 5 **Figure S5.** Fragment bit scores with HMM constructed without QnrS. Bit scores of fragments against the hidden Markov model where all sequences from QnrS were excluded. Click here for file

Additional file 6 **Figure S6.** Specificity. The specificity in classification of fragments of novel qnr genes for each of the five models. The line QnrA denotes the specificity of the model constructed without QnrA to accurately classify fragments from QnrA. The same for QnrB, C, D and S. Click here for file

Additional file 7 **Table S1.** Annotation of the 475 groups of sequences discovered in this work. Click here for file

Additional file 8 **Figure S7.** Overview of the pipeline implementation. A flowchart describing the major parts of the pipeline implemented in Python. Click here for file
