# Supplementary figures and images for: A novel method to discover fluoroquinolone antibiotic resistance (qnr) genes in fragmented nucleotide sequences
Source: BMC Genomics. 2012 Dec 11;13:695. doi: 10.1186/1471-2164-13-695 (PMC3543242; doi:10.1186/1471-2164-13-695)

# Fragment bit scores with HMM constructed without QnrA

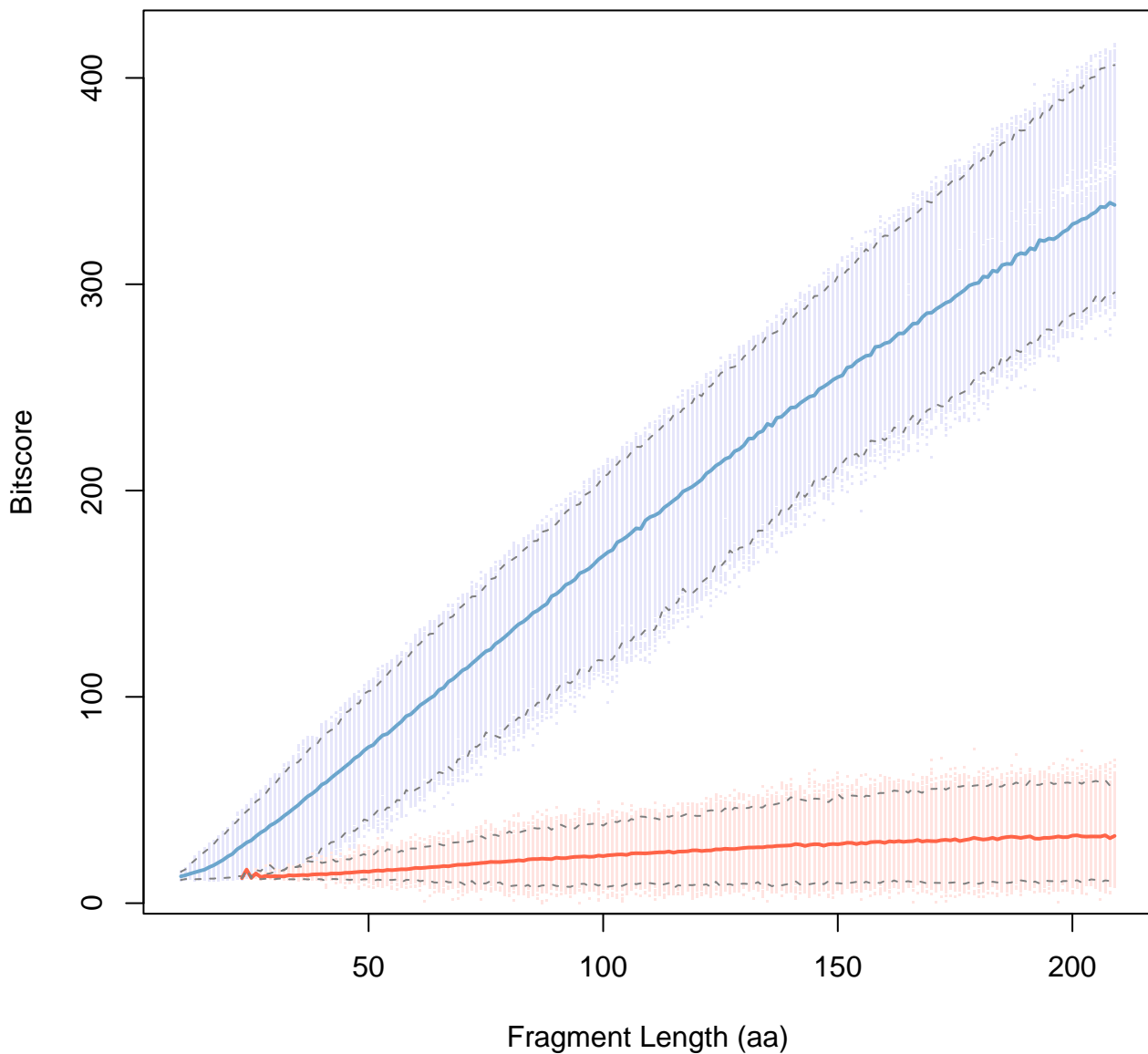

Supplement: Additional file 1 — Figure S1. Fragment bit scores with HMM constructed without QnrA. Bit scores of fragments against the hidden Markov model where all sequences from QnrA were excluded. [file 1471-2164-13-695-S1.pdf]

# Fragment bit scores with HMM constructed without QnrB

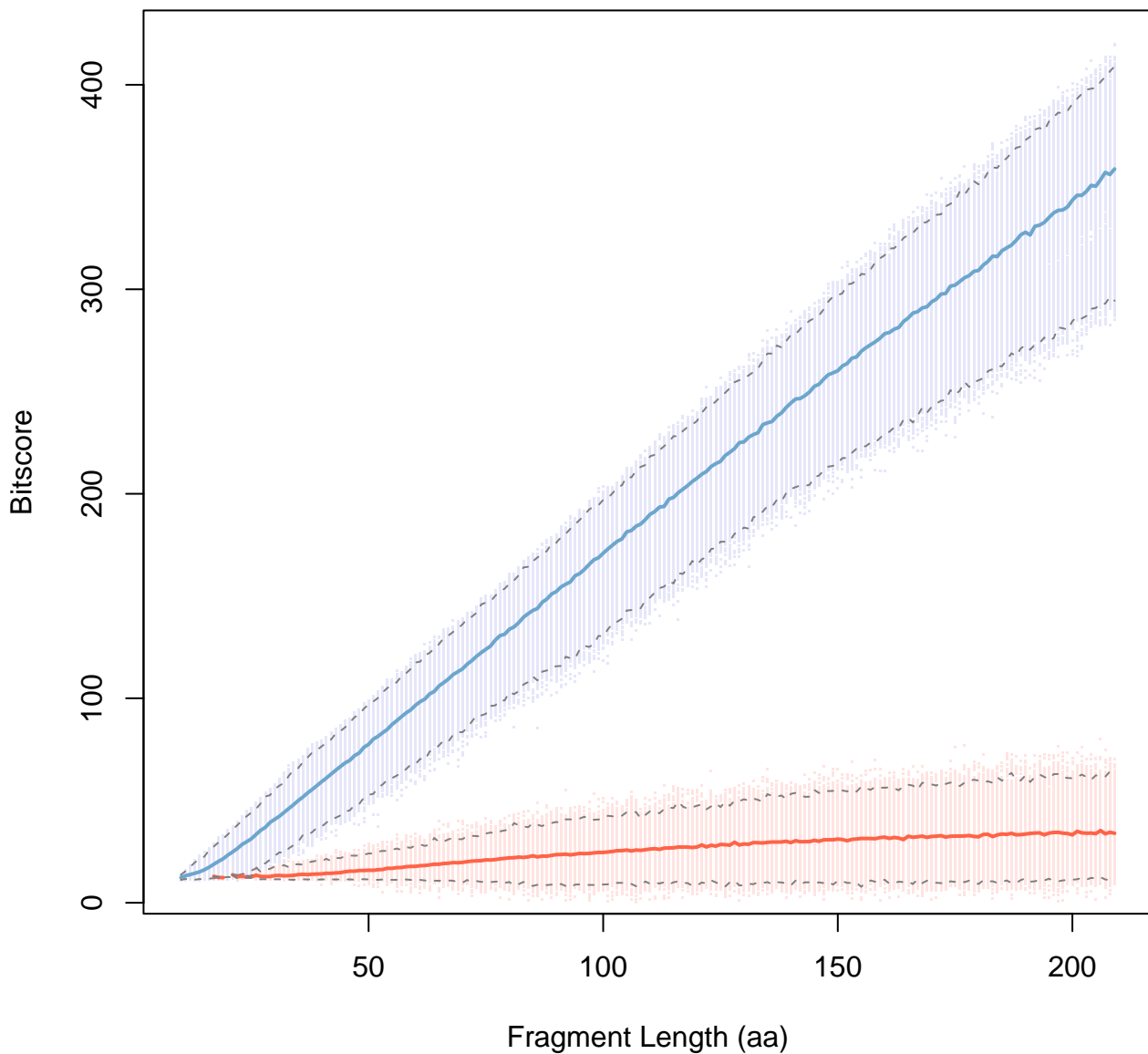

Supplement: Additional file 2 — Figure S2. Fragment bit scores with HMM constructed without QnrB. Bit scores of fragments against the hidden Markov model where all sequences from QnrB were excluded. [file 1471-2164-13-695-S2.pdf]

# Fragment bit scores with HMM constructed without QnrC

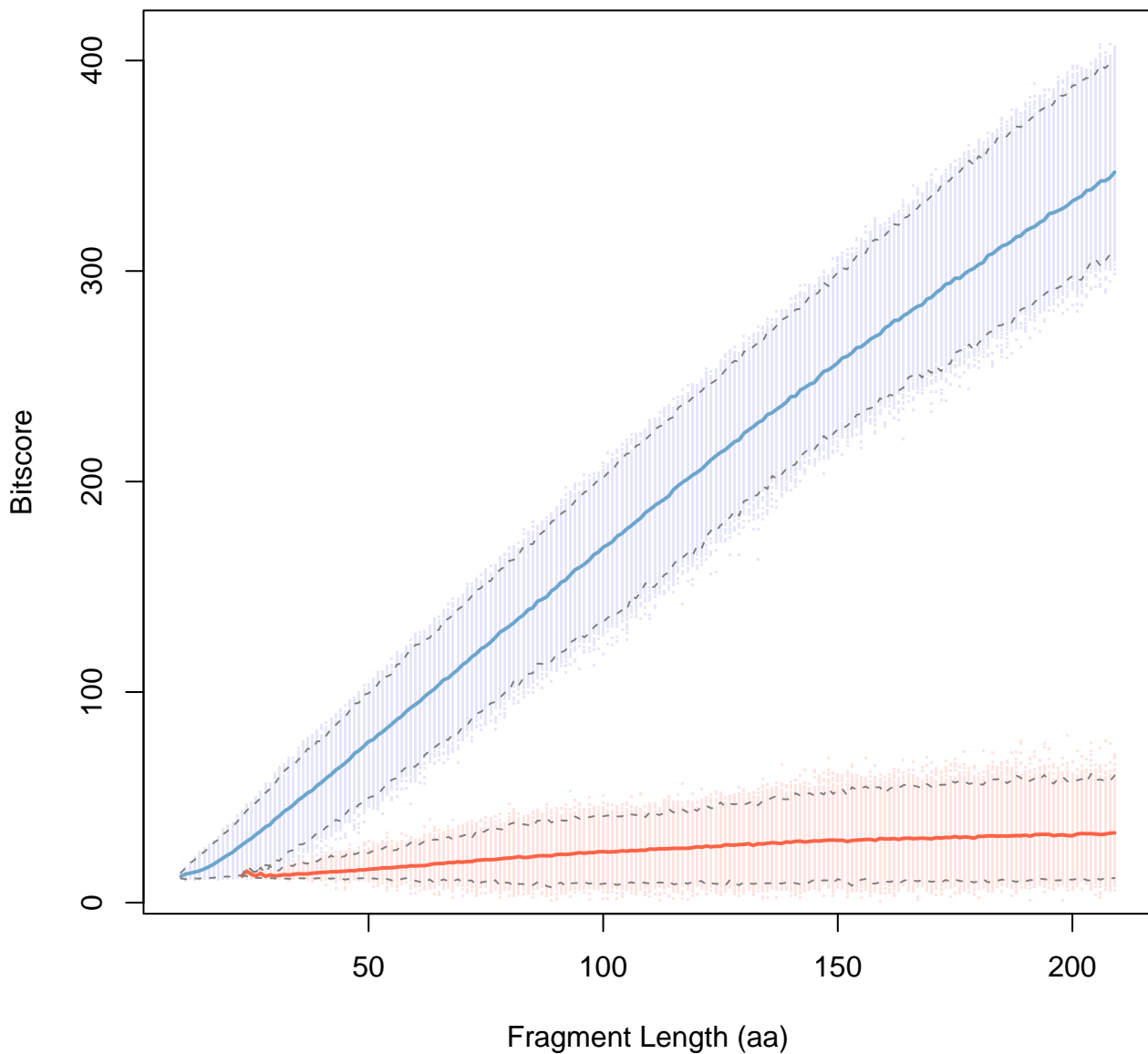

Supplement: Additional file 3 — Figure S3. Fragment bit scores with HMM constructed without QnrC. Bit scores of fragments against the hidden Markov model where all sequences from QnrC were excluded. [file 1471-2164-13-695-S3.pdf]

# Fragment bit scores with HMM constructed without QnrD

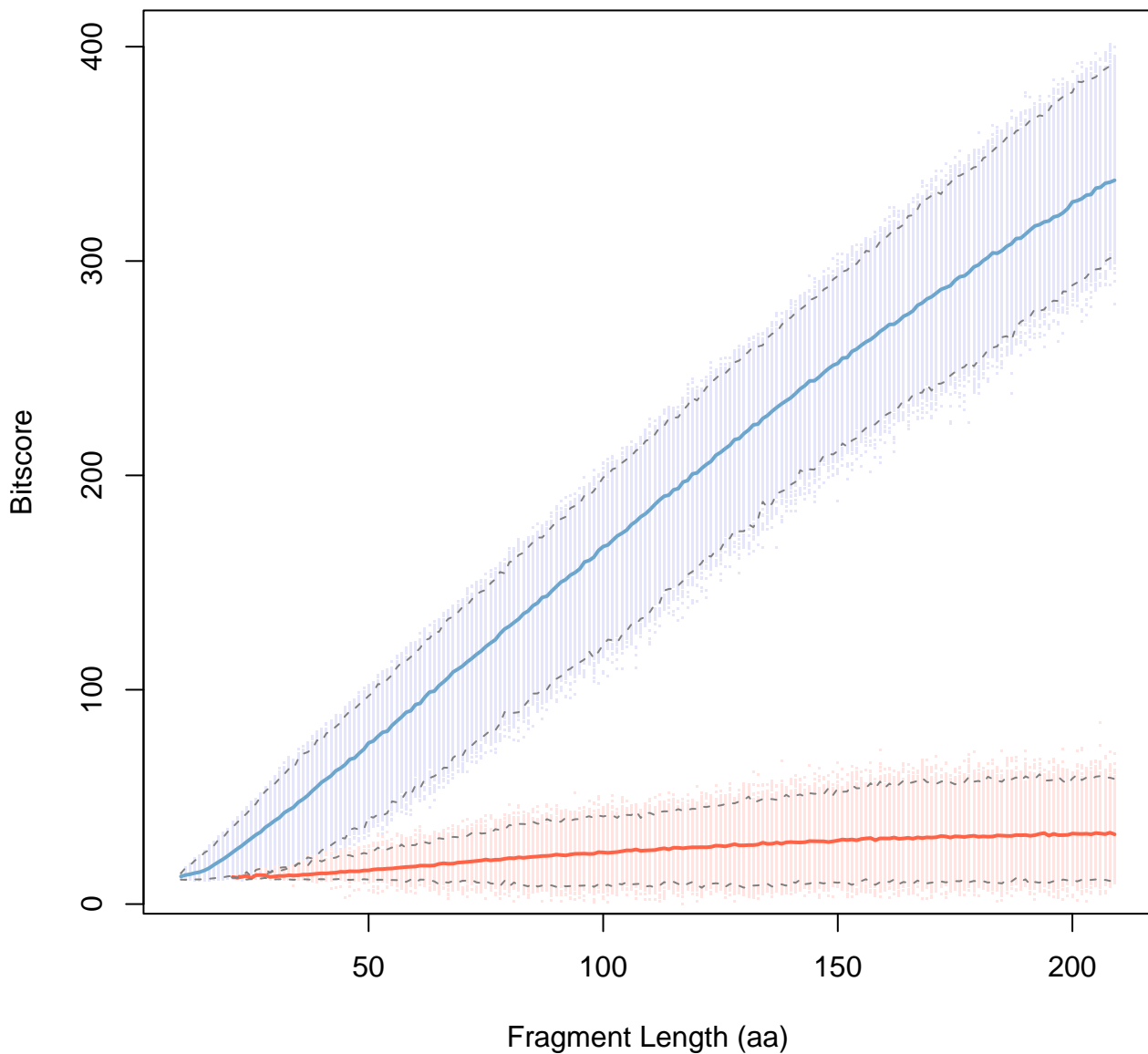

Supplement: Additional file 4 — Figure S4. Fragment bit scores with HMM constructed without QnrD. Bit scores of fragments against the hidden Markov model where all sequences from QnrD were excluded. [file 1471-2164-13-695-S4.pdf]

# Fragment bit scores with HMM constructed without QnrS

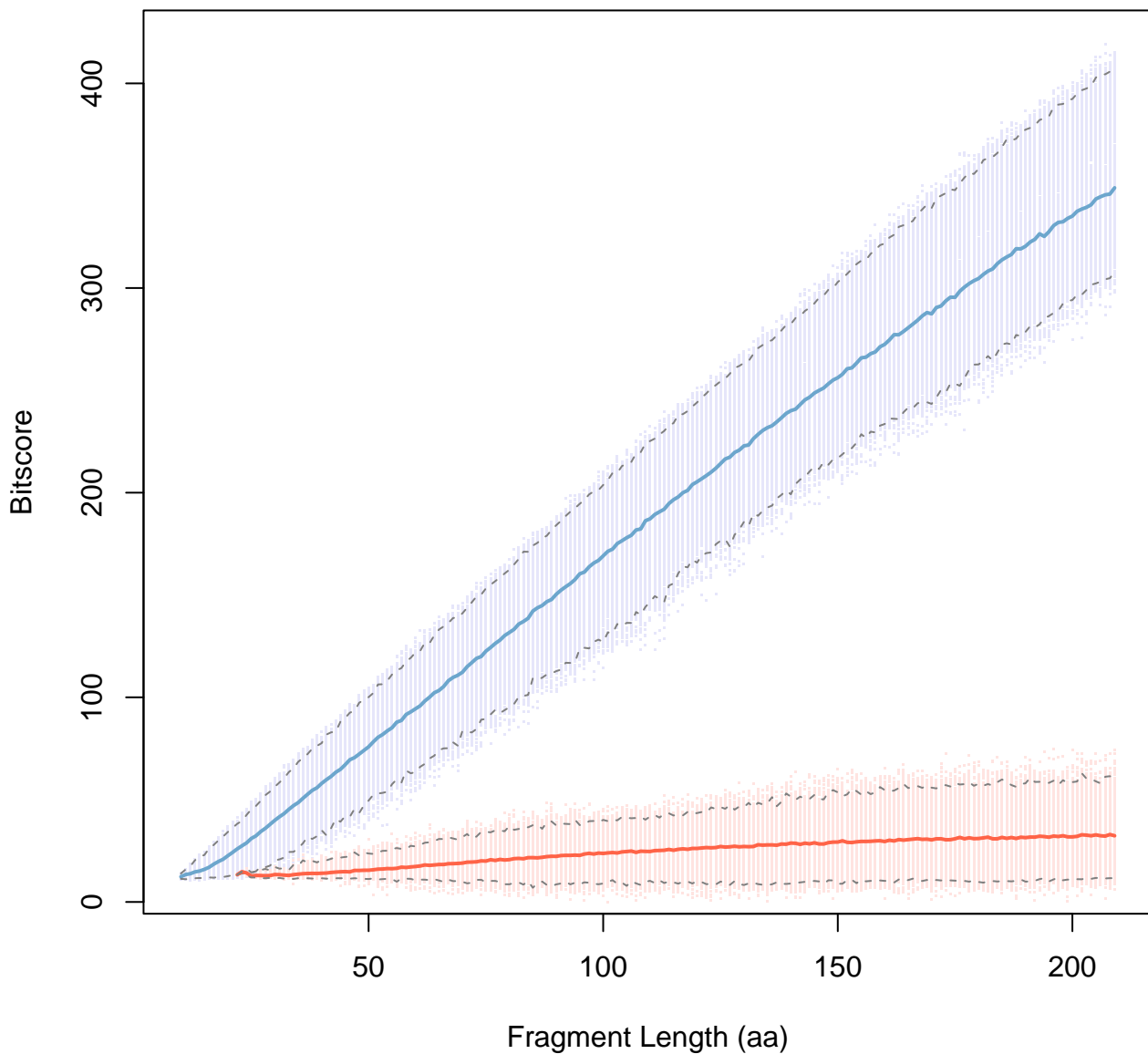

Supplement: Additional file 5 — Figure S5. Fragment bit scores with HMM constructed without QnrS. Bit scores of fragments against the hidden Markov model where all sequences from QnrS were excluded. [file 1471-2164-13-695-S5.pdf]

### Specificity

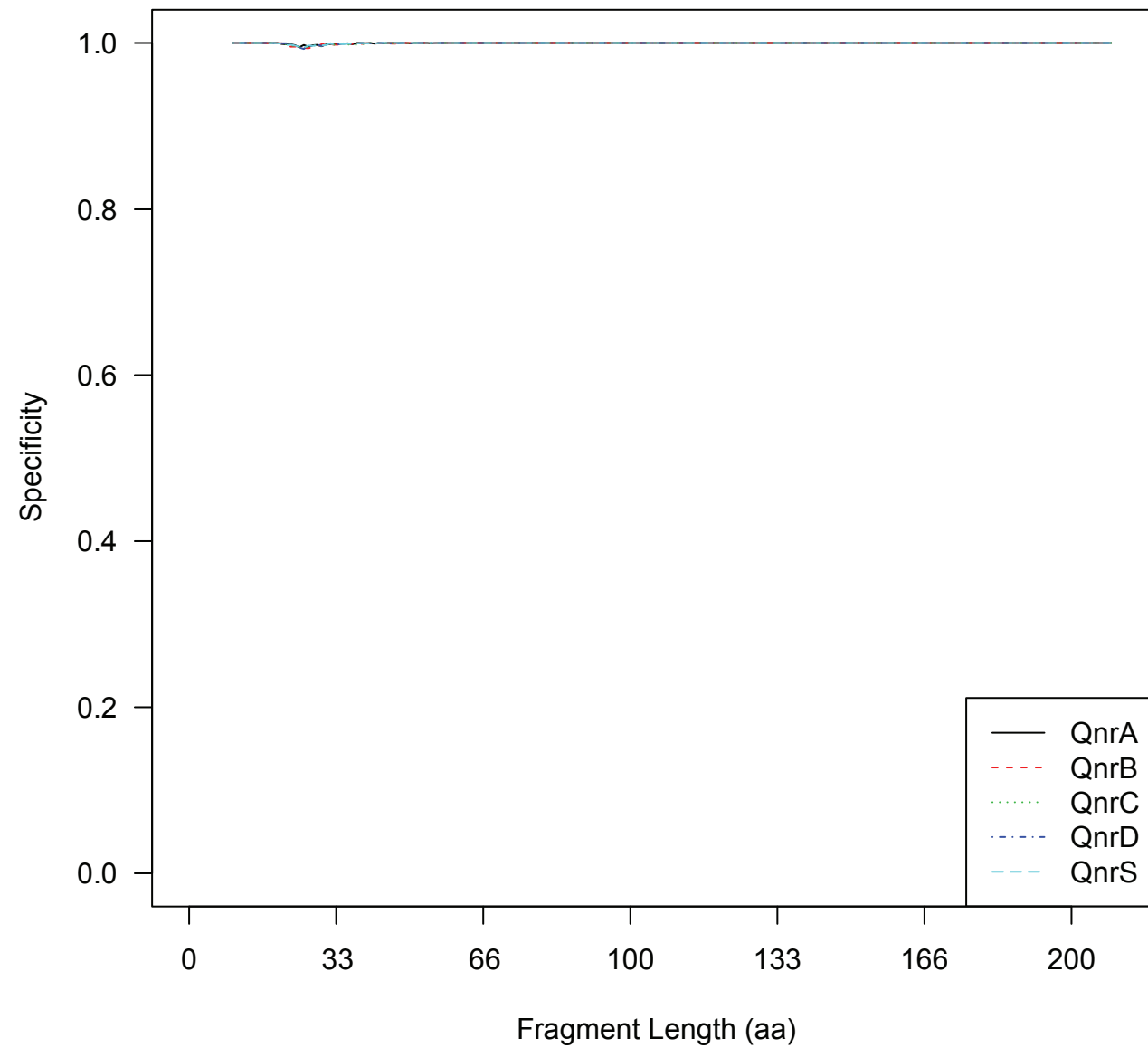

### Specificity (detail)

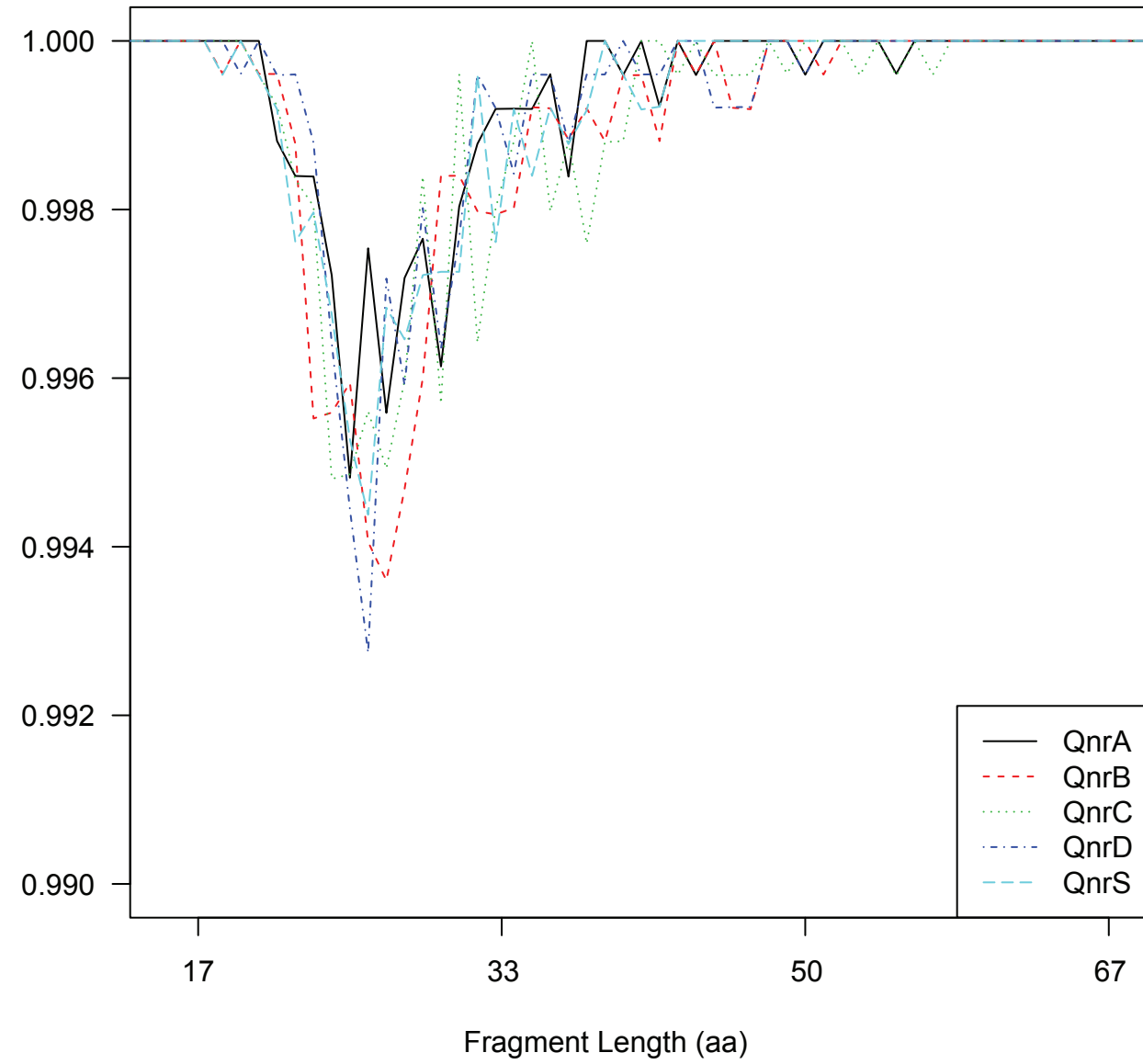

Supplement: Additional file 6 — Figure S6. Specificity. The specificity in classification of fragments of novel qnr genes for each of the five models. The line QnrA denotes the specificity of the model constructed without QnrA to accurately classify fragments from QnrA. The same for QnrB, C, D and S. [file 1471-2164-13-695-S6.pdf]

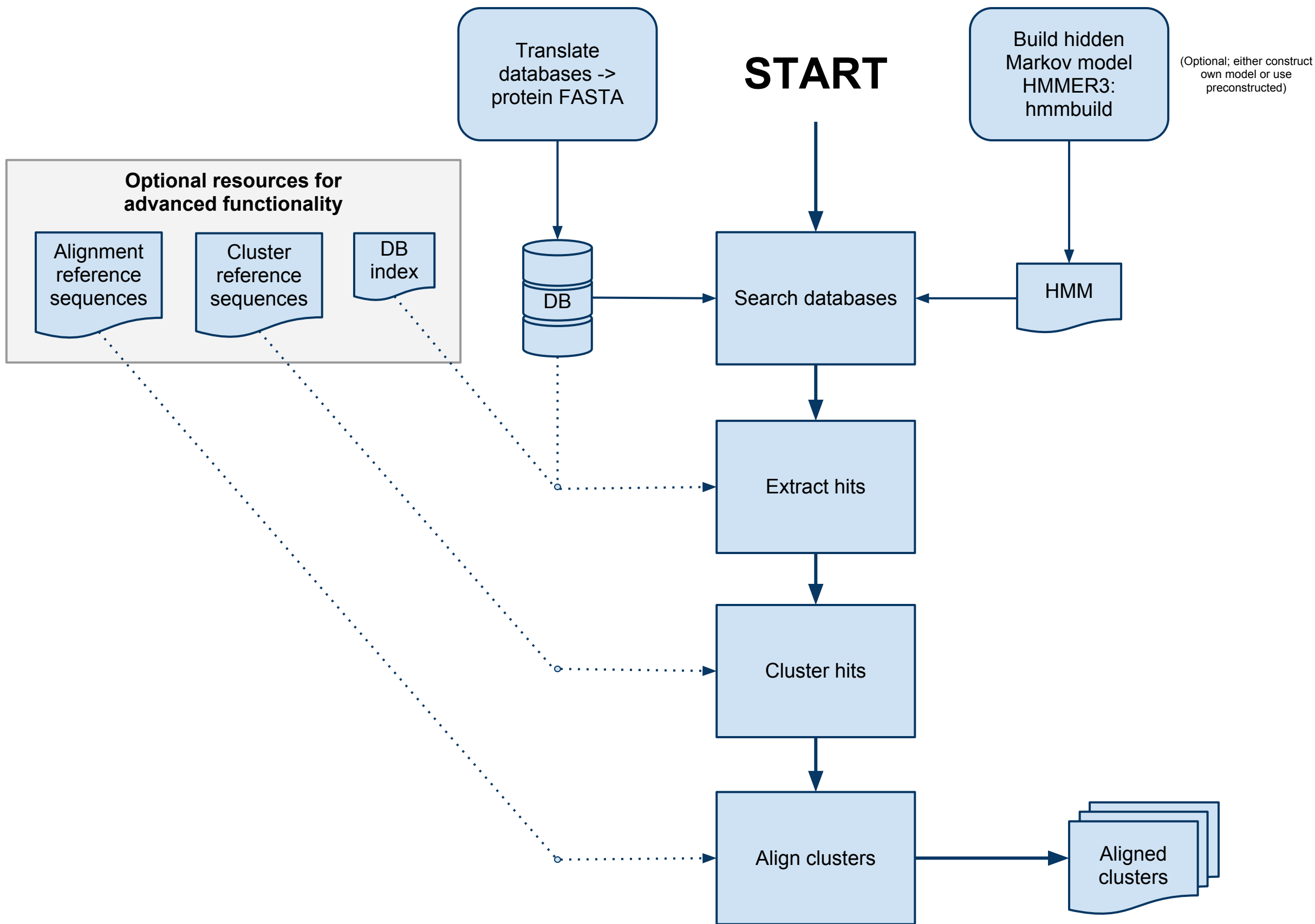

Supplement: Additional file 8 — Figure S7. Overview of the pipeline implementation. A flowchart describing the major parts of the pipeline implemented in Python. [file 1471-2164-13-695-S8.pdf]
